# Improvements in vascular health by a low-fat diet, but not a high-fat diet, are mediated by changes in adipocyte biology

**DOI:** 10.1186/1475-2891-10-8

**Published:** 2011-01-20

**Authors:** Krista A Varady, Surabhi Bhutani, Monica C Klempel, Shane A Phillips

**Affiliations:** 1 Department of Kinesiology and Nutrition, University of Illinois, Chicago, Chicago, IL, USA; 2 Department of Physical Therapy, University of Illinois, Chicago, Chicago, IL, USA

## Abstract

**Background:**

Low-fat (LF) and high-fat (HF) weight loss diets improve brachial artery flow-mediated dilation (FMD) in obese individuals, although results are conflicting. Moreover, the role that adipose tissue plays in mediating these diet-related effects are unknown.

**Objective:**

This study examined how modulations in FMD by HF and LF diets relate to changes in adipocyte parameters.

**Design:**

Obese subjects (n = 17) were randomized to a HF diet (60% kcal as fat) or a LF diet (25% kcal as fat) for 6 weeks. Both groups were restricted by 25% of energy needs.

**Results:**

Body weight decreased (*P <*0.05) in both groups (HF: -6.6 ± 0.5 kg, LF: -4.7 ± 0.6 kg). Fat mass and waist circumference were reduced (*P <*0.05) in the LF group only (-4.4 ± 0.3 kg; -3.6 ± 0.8 cm, respectively). FMD improved (*P <*0.05) in the LF group (7.4 ± 0.8% to 9.8 ± 0.8; 32% increase) and was impaired in the HF group (8.5 ± 0.6% to 6.9 ± 0.7; 19% reduction). Increases in plasma adiponectin (*P <*0.05, 16 ± 5%), and decreases in resistin (*P <*0.05, -26 ± 11%), were shown by the LF diet only. Greater decreases in leptin were observed with LF (-48 ± 9%) versus HF (-28 ± 12%) (*P <*0.05, diet × time). Increased FMD by the LF diet was associated with increased adiponectin, and decreased fat mass, waist circumference, leptin, and resistin.

**Conclusion:**

Beneficial modulations in vascular health by LF diets may be mediated by improvements in adipocyte parameters.

## Introduction

Obesity is a key risk factor for the development of coronary heart disease (CHD). Patients who are obese often exhibit increased LDL cholesterol and triglyceride concentrations, reduced HDL cholesterol concentrations, and increased blood pressure [1]. Carrying this extra weight also leads to abnormal endothelial function marked by reduced vasodilation to an increased blood flow (endothelium-dependent flow-mediated dilation; FMD) [2]. Endothelial dysfunction is an early hallmark and prognostic indicator of future CHD, and most vascular disease risk factors are associated with reduced FMD [3].

Weight loss has been shown to improve plasma lipids, blood pressure, and endothelial function in obese subjects [4]. However, some controversy exists as to the optimal macronutrient composition of the weight loss diet to improve vascular health. For instance, in a study by Phillips et al. [5], 6 weeks of a calorie-restricted high-fat (HF), low carbohydrate diet (60% of kcal as fat, 25% kcal as saturated fat) impaired FMD, while an isocaloric low-fat diet (LF) (25% of kcal as fat, 7% kcal as saturated fat) was shown to improve FMD. In contrast, Volek et al. [5], report increases in FMD with a HF diet, and decreases with a LF diet, while Keogh et al. [6,7] demonstrate no effect of either diet on vascular endothelial function during weight loss. In view of these contradictory findings, more work in this area is evidently required before solid conclusions can be reached.

The mechanisms that link weight loss to improvements in endothelial function are still unclear. However, recent evidence suggests that adipose tissue physiology (i.e. adipokine profile and visceral adiposity) may play a role. Adiponectin is a fat cell-derived hormone that protects the endothelium by decreasing oxidative stress [8]. This hormone has been shown to increase with weight loss and is inversely related to waist circumference (indirect measure of visceral fat mass) [9]. Leptin and resistin, in contrast, are pro-atherogenic hormones derived from adipocytes that have been shown to cause endothelial dysfunction by promoting oxidative stress [10,11]. Leptin and resistin decrease with weight loss and are positively correlated to visceral fat mass [9]. As such, weight loss interventions that reduce visceral fat mass generally result in increased plasma adiponectin, and decreased leptin and resistin. This favorable adipokine profile has a protective effect on the vascular endothelium, which results in increased FMD [12]. An important question that has yet to be tested is whether the enhancements in FMD that occur with either LF or HF diets during weight loss, are mediated in part by improvements in adipocyte parameters.

Accordingly, the objective of this study was to examine the intermediate role of adipocyte parameters in mediating the effects of HF versus LF diets on vascular health during weight loss.

## Subjects and methods

### Subjects

**Figure 1:**
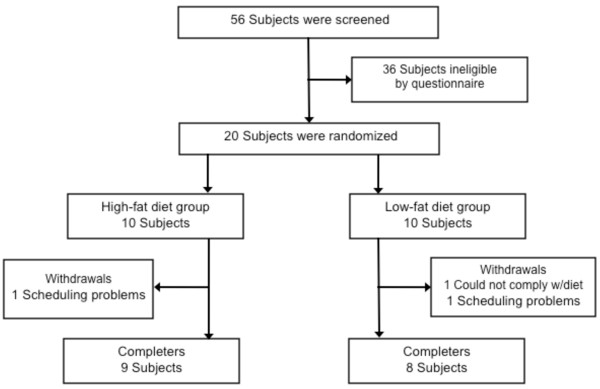
**Study flow chart**.

The study flow chart detailing subject screening, randomization and withdrawals is displayed in Figure 1. Subjects were recruited by means of flyers. Key inclusion criteria were as follows: age 18 to 50 years; body mass index (BMI) between 30 and 39.9 kg/m^2^; normocholesterolemic (defined as total cholesterol concentrations below 200 mg/dl); non-smoking; free of cardiovascular disease; non-diabetic; no history of disordered eating; not dieting; no weight gain or loss > 6 kg in the 3 months prior to the study; not taking lipid or glucose lowering medications; no history of elevated blood pressure (defined as systolic blood pressure >150 mm Hg; diastolic blood pressure >90 mm Hg); and not pregnant or lactating. In addition, peri-menopausal women were excluded from the study, and post-menopausal women (defined as an absence of menses for > 2 years) were required to maintain their current hormone replacement therapy regimen for the duration of the study. The experimental protocol was approved by the Office for the Protection of Research Subjects at the University of Illinois, Chicago. All volunteers gave their written informed consent to participate in the trial prior to the commencement of the study.

### Experimental design

A 6-week randomized, parallel-arm, dietary intervention trial was implemented to test the study objectives. Subjects were randomized to 1 of 2 dietary weight loss groups: 1) HF diet, or 2) LF diet. Random assignment was accomplished with a 1:1 treatment assignment ratio using an envelope-based randomization. Envelopes containing diet assignments were prepared by non-study personnel, sequentially numbered, and identically sealed.

### High-fat and low-fat weight loss diet protocols

All foods and beverages were prepared in the metabolic kitchen of the Clinical Research Center (CRC) and distributed to participants 3 times per week. Subjects in both groups were restricted by 25% of their baseline energy needs. Resting energy expenditure (REE) was measured after a 12-h fast by indirect calorimetry using a metabolic monitor as previously described [13]. The calorie-reduced diets comprised solid foods, typical of those consumed in North America, and were provided as a 3-d rotating menu. All participants were provided with a daily multivitamin and mineral tablet throughout the duration of the study. The macronutrient and dietary composition of each diet is reported in Table 1.

**Table 1:** Macronutrient and dietary composition of provided calorie-reduced diets ^1^

Macronutrient composition	HF diet	LF diet
Energy (kcal)	1868 ± 106	1967 ± 93
Carbohydrate (% kcal)	5 ± 1	55 ± 2
Protein (% kcal)	35 ± 1	20 ± 1
Protein-energy ratio (g/100 kcal)	8.7 ± 1	4.8 ± 1
Fat (% kcal)	60 ± 1	25 ± 1
Saturated fat (% kcal)	26 ± 1	7 ± 1
Monounsaturated fat (% kcal)	24 ± 1	9 ± 1
Polyunsaturated fat (% kcal)	9 ± 1	9 ± 1
Trans fat (% kcal)	1 ± 0	0 ± 1
Cholesterol (mg)	448 ± 14	299 ± 3
Fiber (g)	11 ± 2	30 ± 2

Dietary composition	HF diet	LF diet

Breakfast	Eggs	Oatmeal
	Ham	Canned peaches
Lunch	Lettuce w/carrots and cucumber	Lettuce w/carrots and cucumber
	Roast beef	Tuna
	Cheddar cheese	Walnuts
	Olive oil/vinegar dressing	Ranch dressing
Dinner	Chicken provencal	Macaroni with beef
	Potatoes w/butter	Dinner roll
	Broccoli	Broccoli

^1 ^Data are presented as mean ± SEM. HF: high fat, LF: low fat.

### Dietary compliance and physical activity assessment

The subjects were instructed to eat only the foods provided and to abstain from alcohol throughout the study. Compliance with the experimental diet was assessed by means of daily recording of added and missed foods from the menu plan. If the deviations averaged > 5% of total calories each day, the subject was considered noncompliant and the data were not included in the analyses. Dietary compliance was also assessed by measuring the ratio of urinary urea to creatinine. A 24-h urine sample was collected at week 1 and 6 for the assessment of sodium excretion and urea/creatinine ratio. The urine volume was recorded and aliquots were frozen until analysis. Urea and creatinine were measured using enzymatic kits (Roche Diagnostics, Indianapolis, IN). Subjects were also instructed to maintain their customary level of physical activity throughout the duration of the study. An electronic pedometer was used for the quantitative measurement of changes in physical activity. Study participants were instructed (including a demonstration) how to use the pedometer properly, according to the manufacturer's instructions. All study participants wore the pedometer each day of the 6-week study, and were instructed to record step counts in a log at the end of each day.

### Blood collection protocol

Twelve-hour fasting blood samples were collected at week 1 (day 1) and at week 6 (day 42). Blood was centrifuged for 15 min at 520 × g and 4°C to separate plasma from red blood cells, and was stored at -80°C.

### Body weight and body composition assessment

Body weight was assessed in the fasted state without shoes and in light clothing at week 1 and 6, using a balance beam scale (Health-O-Meter, Terre Haute, IN). Height was measured using a wall-mounted stadiometer. BMI was calculated as kg/m^2^. Fat mass and fat free mass were assessed in triplicate after each weigh-in using a tetra-polar bioelectrical impedance analyzer (Xitron, San Diego, CA). The instrument recorded impedance from hand to foot and consequently, calculated fat mass and fat free mass from the impedance value and the pre-entered personal particulars (weight, height, age and sex). The coefficient of variation within run for percent body fat was 3.1%. Waist circumference was measured in triplicate in standing subjects as the minimum abdominal circumference between the xyphoid process and the umbilicus [14].

### Brachial artery measurements of flow-mediated dilation (FMD)

Brachial artery FMD was assessed at week 1 and 6. Ultrasound imaging of the brachial artery (Logiq 500 Pro Ultrasound System, General Electric COmpany, Schenectady, NY) was performed in a longitudinal plane at a site 1-3 cm proximal to the antecubital fossa, with the arm abducted approximately 80° from the body and the forearm supinated. The ultrasound probe (11 MHz) was positioned to visualize the anterior and posterior lumen-intima interfaces to measure diameter or central flow velocity (pulsed Doppler). After baseline images were recorded, a blood pressure cuff on the forearm was inflated to 200 mm Hg for 5 min. To assess FMD, 10 seconds of images were captured at a rate of 10 images/second, 30 seconds, 1 min, and 2 min after cuff release. Images were digitally recorded using Brachial Imager (Medical Imaging, Iowa City, Iowa) at 10 frames per second for 10 seconds at each time period. Brachial artery diameter was measured from the intimal medial border interface on each frame using the average of 100 distinct evenly spaced longitudinal diameters for each measurement using an automated edge-detection algorithm. The minimum (diastolic) diameters were determined for each cardiac cycle and these minimum diameters (n = 8-10) were then averaged to obtain one brachial artery diameter at each time point. Percent FMD was calculated using the averaged minimum mean brachial artery diameter at baseline compared to the largest mean values obtained after either release of the forearm occlusion. Percent FMD was calculated using the averaged minimum mean brachial artery diameter at baseline compared to the largest mean values obtained after release of the forearm occlusion.

### Plasma adipokine determination

Plasma adiponectin, leptin, and resistin concentrations were quantified using high sensitivity ELISA kits (R&D Systems, Minneapolis, MN). Intra-assay precision of the adiponectin, leptin, and resistin ELISA kits were 4.1%, 3.8%, and 4.2%, respectively.

### Cardio-metabolic risk factor assessment

Total cholesterol, direct LDL cholesterol, HDL cholesterol, and triacylglycerol concentrations were measured using enzymatic kits (Roche Diagnostics, Indianapolis, IN), with intra-assay variances of 3.7%, 3.7%, 4.1%, and 2.6%, respectively. Blood pressure was measured after a 5-min rest after the weigh-in. C-reactive protein (CRP) was measured by ELISA, (R&D Systems Inc., Minneapolis, MN), with an intra-assay variance of 3.0%. Plasma glucose concentration was measured using the glucose oxidase procedure (Beckman II autoanalyzer), and plasma insulin was measured by radioimmunoassay (Linco Research, MO), with intra-assay variances of 3.8% and 4.3%, respectively.

### Statistical analysis

Data are presented as mean ± SEM. All variables were examined for a normal distribution, and abnormally distributed variables were log-transformed. One-factor analysis of variance was used to compare baseline characteristics. The effect of the dietary intervention was assessed by using repeated-measures analysis of variance, with time as the within-subject factor and diet (HF compared with LF) as the between-subjects factor. Post hoc comparisons were performed by Tukey's honestly significant difference test. Correlation analysis was used to determine relationships between variables. Statistical significance was set at *P *< 0.05. Data were analyzed using SPSS software (version 18.0; SPSS Inc, Chicago, IL).

## Results

### Subject baseline characteristics and dropouts

Twenty subjects commenced the study, with 17 completing the entire 6-week trial (HF group, n = 9; LF group, n = 8) (Figure 1). Two subjects dropped out due to scheduling problems, while one other dropped out due to inability to comply with the LF diet protocol. No significant differences in baseline variables were observed between groups (Table 2).

**Table 2:** Baseline characteristics of subjects who completed the 6-week trial ^1,2^

	HF diet (n = 9)	LF diet (n = 8)
Male	3	2
Female	6	6
Age (y)	35 ± 3	36 ± 3
Body weight (kg)	97 ± 5	98 ± 4
BMI (kg/m^2^)	33 ± 1	34 ± 2
Total cholesterol (mg/dL)	147 ± 11	152 ± 10
LDL cholesterol (mg/dL)	82 ± 10	95 ± 8
HDL cholesterol (mg/dL)	57 ± 5	47 ± 4
Triacylglycerol (mg/dL)	75 ± 14	62 ± 8
Systolic blood pressure (mm Hg)	125 ± 5	131 ± 7
Diastolic blood pressure (mm Hg)	72 ± 3	75 ± 3
Steps per day	9974 ± 2701	10257 ± 2862

^1 ^Data are presented as mean ± SEM.HF: high fat, LF: low fat.

^2 ^No differences between HF and LF group for any baseline characteristic. Data were analyzed using One-factor ANOVA.

### Dietary compliance and physical activity

Adherence to the dietary interventions was established by the ratio of urinary urea to creatinine. The ratio was not different between groups at baseline (HF: 35 ± 4; LF: 31 ± 2, *P = *0.16) but was significantly different between diets at week 6 (HF: 40 ± 2; LF: 31 ± 3, *P = *0.04 for diet × time interaction). Changes in physical activity, assessed by wearing a pedometer each day, were not different between groups throughout the trial (HF: 9754 ± 2256 steps/d; LF: 10090 ± 1842 steps/d, *P = *0.35).

### Changes in body weight and body composition by HF versus LF diets

Body weight was reduced (*P *< 0.05) in both the HF and LF groups by week 6 (Table 3). However, the HF group lost significantly more weight from baseline (-6.6 ± 0.5 kg) than the LF group (-4.7 ± 0.6 kg) (*P <*0.05 for diet × time interaction). BMI decreased (*P <*0.05) in both the HF (-2.0 ± 0.2 kg/m^2^) and LF group (-1.7 ± 0.2 kg/m^2^) after 6 weeks of diet. Fat mass and waist circumference were reduced (*P <*0.05) in the LF group only (-4.4 ± 0.3 kg; -3.6 ± 0.8 cm, respectively). As for fat free mass, decreases from baseline (*P <*0.05) were observed only for the HF group (-3.0 ± 0.2 kg).

**Table 3:** Body weight and body composition during the 6-week trial ^1^

	HF diet (n = 9)		LF diet (n = 8)	
	**Week 1**	**Week 6**	**Week 1**	**Week 6**

Body weight (kg)	97.0 ± 4.5	90.4 ± 4.3^2,3^	98.3 ± 3.9	93.6 ± 4.0^2^
BMI (kg/m^2^)	32.5 ± 1.0	30.5 ± 1.0^2^	34.4 ± 1.5	32.7 ± 1.9^2^
Fat mass (kg)	41.8 ± 1.6	39.5 ± 1.4	41.0 ± 1.5	36.6 ± 1.5^2^
Fat free mass (kg)	53.8 ± 4.0	50.8 ± 3.4^2^	57.3 ± 2.5	56.9 ± 2.5
Waist circumference (cm)	97.3 ± 2.3	95.8 ± 1.9	98.5 ± 2.7	94.9 ± 1.6^2^

^1^Data are presented as mean ± SEM. HF: high fat, LF: low fat. Data were analyzed using repeated-measures ANOVA.

^2^*P <*0.05 for main effect of time.

^3^P < 0.05 for diet × time interaction such that HF group had greater reductions in body weight than the LF group.

### Improvements in vascular endothelial function by HF versus LF diets

**Figure 2:**
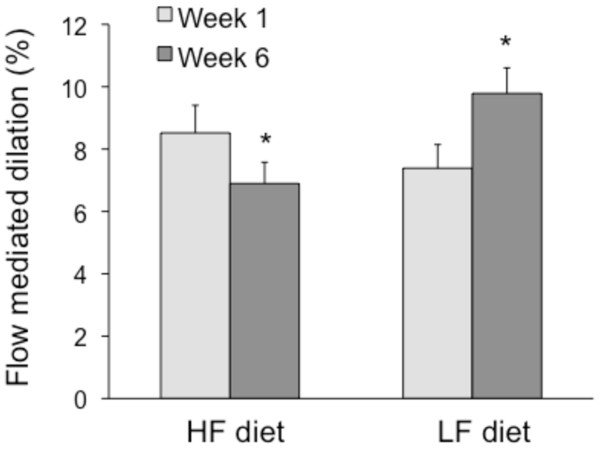
**Flow-mediated dilation during the 6-week trial**. Data are presented as mean ± SEM. HF: high fat, LF: low fat. HF group (n = 9), LF group (n = 8). Data were analyzed using repeated-measures ANOVA. *P <0.05 for main effect of time.

There were no differences in brachial artery FMD between groups at baseline (Figure 2). By week 6, FMD improved (*P <*0.05) in the LF group relative to baseline (7.4 ± 0.8% to 9.8 ± 0.8; 32% increase). In contrast, FMD was impaired (*P <*0.05) in the HF group by week 6 relative to baseline (8.5 ± 0.6% to 6.9 ± 0.7; 19% reduction).

### Plasma adipokine changes by HF versus LF diets

**Figure 3:**
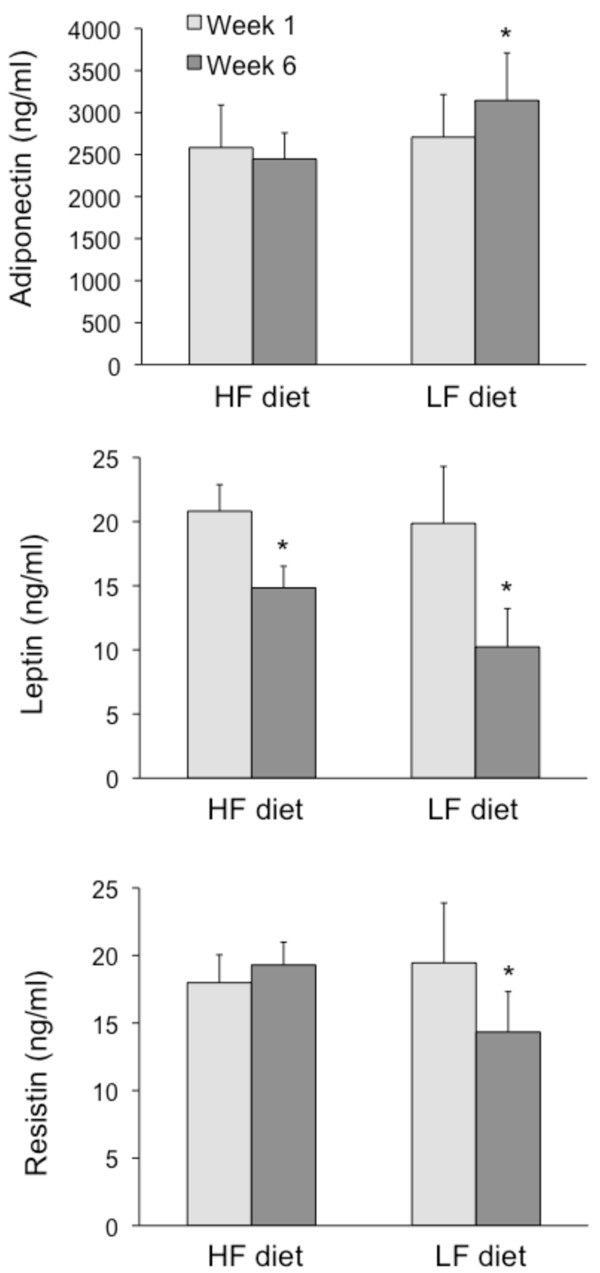
**Plasma adipokines during the 6-week trial**. Data are presented as mean ± SEM. HF: high fat, LF: low fat. HF group (n = 9), LF group (n = 8). Data were analyzed using repeated-measures ANOVA. *P <0.05 for main effect of time.

After 6 weeks of diet, circulating adiponectin increased (*P <*0.05) with weight loss in the LF group (16 ± 5%), but remained unchanged in the HF group (Figure 3). Leptin concentrations were reduced (*P <*0.05) with weight loss in both the HF group (-28 ± 12%) and LF group (-48 ± 9%). However, the LF group experienced greater decreases in leptin from baseline than the HF group (*P <*0.05 for diet × time interaction). Plasma resistin decreased (*P <*0.05) with weight loss in the LF group only (-26 ± 11%).

### Relationship between vascular endothelial function and adipose tissue parameters

After 6 weeks of diet, increased FMD by the LF diet was associated with increased adiponectin, and decreased fat mass, waist circumference, leptin, and resistin (Table 4). There were no significant associations between FMD and any of these adipose tissue variables in the HF group (data not shown).

**Table 4:** Changes in FMD relative to changes in adipose tissue parameters in the LF group at week 6 ^1^

	Δ Body weight	Δ Fat mass	Δ WC	Δ Adiponectin	Δ Leptin	Δ Resistin
FMD	*r = *-0.15	***r *= -057**	***r *= -0.81**	***r *= 0.55**	***r *= -0.78**	***r *= -0.47**
	*P = *0.72	***P *= 0.04**	***P *= 0.01**	***P *= 0.04**	***P *= 0.03**	***P *= 0.01**

^1 ^Δ: percent change from baseline, FMD: flow-mediated dilation, WC: waist circumference.

### Cardio-metabolic risk modulations by HF versus LF diets

Total and LDL cholesterol were lower (*P <*0.05) after 6 weeks of diet in the LF group (Table 5). In contrast, total cholesterol increased (*P <*0.05) with weight loss in HF group, while LDL cholesterol was not affected. Triacylglycerols and HDL cholesterol concentrations were not altered by either intervention. Plasma lipids were not correlated with FMD at baseline or post-treatment. Systolic blood pressure was lower (*P <*0.05) with weight loss in both groups, but decreased more with the LF diet (*P <*0.05 for diet × time interaction). Similar reductions (*P <*0.05) in diastolic blood pressure values were noted for the HF and LF diet. Glucose was not affected by either diet, while insulin decreased (*P <*0.05) in both diet groups. CRP concentrations did not change with either diet.

**Table 5:** Cardio-metabolic risk indicators during the 6-week trial ^1^

	HF diet (n = 9)		LF diet (n = 8)	
	**Week 1**	**Week 6**	**Week 1**	**Week 6**

Total cholesterol (mg/dL)	147 ± 11	162 ± 7^2^	152 ± 10	139 ± 7^2^
LDL cholesterol (mg/dL)	82 ± 10	90 ± 14	95 ± 8	86 ± 10^2^
HDL cholesterol (mg/dL)	57 ± 5	56 ± 5	47 ± 4	40 ± 2
Triacylglycerols (mg/dL)	75 ± 14	57 ± 10	62 ± 8	74 ± 10
SBP (mm Hg)	125 ± 5	114 ± 3^2^	131 ± 8	115 ± 3^2,3^
DBP (mm Hg)	72 ± 3	67 ± 3^2^	75 ± 3	68 ± 4^2^
Glucose (mg/dL)	92 ± 1	96 ± 2	90 ± 2	91 ± 2
Insulin (mg/dL)	19 ± 3	13 ± 1^2^	18 ± 5	14 ± 2^2^
CRP (mg/dL)	6 ± 1	7 ± 1	5 ± 2	5 ± 2

^1^Data are presented as mean ± SEM. CRP: C-reactive protein, DBP: diastolic blood pressure, HF: high fat, LF: low fat, SBP: systolic blood pressure. Data were analyzed using repeated-measures ANOVA.

^2^*P <*0.05 for main effect of time.

^3^*P <*0.05 for diet × time interaction such that LF group had greater reductions in systolic blood pressure than the HF group.

## Discussion

This study is the first to show that improvements in endothelial function by LF diets during weight loss may be mediated in part by changes in adipose tissue biology. More specifically, we show here that improved brachial artery FMD during LF diets is associated with decreased visceral fat mass and improved adipokine profile (i.e. increased plasma adiponectin, and decreased leptin and resistin). These benefits of LF diets on vascular health were paralleled by improvements in several CHD risk factors. In contrast, weight loss by HF diets, were shown to impair FMD, with no concomitant improvement in parameters of adipocyte biology.

Brachial artery FMD is a noninvasive index of endothelial function [15], and is an independent predictor of cardiovascular events [16]. Diet-induced weight loss has been shown to be an effective strategy to help obese individuals increase FMD, however, the optimal macronutrient composition of the diet remains unknown. While some studies indicate that LF diets may benefit vascular health more than HF diets [17,18], other studies show that HF diets are superior [5]. In the present study, we demonstrate that weight loss resulting from an LF diet improves FMD, while weight loss resulting from a HF diet impairs FMD. Although these findings are similar to that of Phillips et al. [17], they are in contrast to the results of Keogh et al. [6,7] and Volek et al. [5]. The reason for these conflicting data remain unclear since trial design in each study was similar with respect to sample population, trial length, and dietary macronutrient distribution. One key difference, however, is the dietary protocols used. In our study, as well as in the study by Phillips et al. [17], all meals were provided to the subjects so that macronutrient distribution would be carefully controlled. In the trials by Keogh et al. [6,7] and Volek et al. [5], subjects were instructed how to consume the HF or LF diets by a dietician, and dietary adherence was assessed by means of food records. Since macronutrient distribution was not carefully controlled in the studies by Keogh et al. [6,7] and Volek et al. [5], it is possible that the subjects were consuming something closer to a standard diet (i.e. 50% kcal as carbohydrate, 15% kcal as protein, and 35% kcal as fat), rather than a HF or LF diet. As such, the lack of standardization of the feeding protocols may partly explain these inconsistent findings. The reason why the HF diet impaired FMD is not certain, but may involve the increased consumption of saturated fat. Subjects in the HF group consumed 26% of energy as saturated fat, which is similar to that consumed with Atkins'-like diets. Evidence suggests that a high intake of saturated fat directly impairs arterial endothelial function by reducing the anti-inflammatory potential of HDL [19]. Therefore, the increased saturated fat intake may have contributed to the decreased FMD observed in the HF group. It should also be noted that the diets varied greatly in dietary fiber content (HF diet: 11 g/d, LF diet: 30 g/d). Recent findings by Rallidis et al. [20] indicate that increasing fiber consumption may augment FMD in obese volunteers. As such, this discrepancy in dietary fiber content between the two groups may have contributed to the beneficial alterations in FMD observed by the LF diet, but not the HF diet.

The differences in endothelial function observed between HF and LF groups may also be related to changes in body composition. We show here that HF subjects lost more weight (-6.6 ± 0.5 kg) than LF subjects (-4.7 ± 0.6 kg). However, fat mass and waist circumference were significantly decreased in LF subjects only. It was also observed that the decreases in fat mass and waist circumference by LF diets were correlated to improvements in FMD post-treatment. Accumulating evidence suggests that visceral fat mass is inversely correlated to FMD [21]. A mechanism linking visceral adiposity to the onset and progression of endothelial dysfunction may involve free fatty acids (FFA) [22]. In vitro studies demonstrate that excessive release of FFA by visceral fat into the bloodstream can enhance the production of reactive oxygen species, which may induce endothelial dysfunction [23]. In view of these relationships, it is possible that the decrease in visceral fat mass in the LF group contributed to the improvements in FMD observed here. It is important to note, however, that this study is limited in that waist circumference was used to assess visceral fat mass. Waist circumference is only an indirect indicator of visceral adiposity, and is strongly operator dependent. As such, future studies in this area should implement computed tomography (CT) scans to distinguish between visceral and subcutaneous fat mass, and to confirm the association of visceral adiposity with FMD.

Favorable changes in adipokine profile may have also played a role in the improvements in FMD observed with LF diets. After 6-weeks of treatment, subjects in the LF group experienced a 16% increase in plasma adiponectin from baseline, which was paralleled by a 48% and 26% decrease in leptin and resistin concentrations, respectively. We also observed that the increases in adiponectin, and decreases in leptin and resistin were associated with improved FMD. Although a causal link between adiponectin and FMD has yet to be established, we speculate that modulations in nitric oxide (NO) by adiponectin may be involved. Nitric oxide, released from the endothelium, is important in regulating vascular tone. Plasma adiponectin can stimulate the phosporylation of endothelial nitric oxide synthase (eNOS), thereby increasing NO-dependent endothelial vasodilation [24]. Therefore, higher circulating concentrations of adiponectin in the LF group may contribute to enhanced endothelial function. As with adiponectin, we hypothesize that the mechanism linking decreased leptin and resistin to increased FMD may involve changes in the production of NO. Both leptin and resistin have been shown to blunt NO production [11,25] likely through the stimulation of reactive oxygen species that scavenge NO and impair eNOS function. Since these adipokines were significantly decreased post-intervention in the LF group, there would be less leptin and resistin in the circulation to inhibit NO. Therefore, more NO may have been produced, resulting in an enhancement in endothelium-dependent vasodilation. Visceral fat mass loss is a major determinant of adipokine profile improvement with diet [26]. In the present study, significant reductions in visceral fat mass were noted in the LF group, but not the HF group. As such, it can be hypothesized that this reduction in abdominal fat mass by the LF regimen contributed to the increases in adiponectin, and decreases in leptin and resistin observed.

Several previous studies have evaluated the effects of HF and LF energy restricted diets on cardiometabolic disease risk factors [5-7,17]. In general, both of these diets have been shown to improve blood pressure, CRP, glucose, and insulin concentrations [5-7,17]. We show here that HF and LF diets were equally as effective in reducing blood pressure (systolic and diastolic) and insulin. However, differential effects for each diet on plasma lipids were observed. For instance, in the LF group, total and LDL cholesterol concentrations were reduced post-intervention. In contrast, in the HF group, total cholesterol *increased *while LDL cholesterol remained unchanged. These effects of HF diets on cholesterol concentrations are in line with previous findings [27]. Diets that are high in saturated fat are thought to increase total and LDL cholesterol concentrations by affecting LDL receptor activity, protein, and mRNA abundance [28]. These mechanisms may therefore account for the increased total cholesterol concentrations observed with HF diets.

There are several limitations to our study that warrant discussion. Firstly, our study is limited in that it employed a small sample size (HF diet: n = 9, LF diet: n =8). This imposes risk of a type 2 error, and as such, non-significant results may be merely the result of low statistical power. An example of this would be the lack of significant difference for FMD at baseline between groups. If a larger sample size was employed, it can be postulated that a significant difference in FMD between groups at this time point would be observed. Secondly, our experimental design is limited in that no control group was implemented. The omission of a control group makes it difficult to ascertain whether these effects were truly due to treatment, and not the result of an unidentified confounder. Future studies in this area should include a control group to ensure that these effects can be solely attributed to diet. Thirdly, our findings cannot be generalized to *all *HF and LF diets. Since not all HF and LF diets are created equal, it is possible that these effects may have occurred in response to the specific diets and food items provided here.

In sum, our findings suggest that weight loss with LF diets may be superior to that of HF diets for improving endothelial function and cardiometabolic risk variables. Our results also show that these vascular benefits of LF diets may be mediated, in part, by improvements in adipose tissue parameters (i.e. body fat distribution and adipokine profile). Further research is warranted to determine the impact of both dietary patterns on long-term cardiovascular outcomes, and the role that adipose tissue may play in mediating these effects.

## Competing interests

The authors declare that they have no competing interests.

## Authors' contributions

KAV performed the statistical analysis and wrote the manuscript. SB and MCK were involved with the laboratory analysis, helped with the interpretation of the data, and assisted with manuscript preparation. SAP designed the study, oversaw study recruitment, performed vascular analysis, and assisted with the preparation of the manuscript. All authors read and approved the final manuscript.
